# Integration across sectors: Canada’s national action plan on criminal justice and mental health

**DOI:** 10.3389/fpubh.2026.1960661

**Published:** 2026-10-08

**Authors:** Christopher Canning, Laura Justine Chouinard, Sandra Koppert, Laura Mullaly, Sierra Slaunwhite, Katie Ellis, Howard Sapers

**Affiliations:** 1 Waypoint Research Institute, Waypoint Centre for Mental Health Care, Penetanguishene, ON, Canada; 2 Department of Psychiatry, University of Toronto, Toronto, ON, Canada; 3 Mental Health Commission of Canada, Ottawa, ON, Canada; 4 Canadian Civil Liberties Association, Ottawa, ON, Canada

**Keywords:** action plan, continuity of care, correctional mental healthcare, discharge planning, forensic mental healthcare, healthcare governance, information sharing

## Abstract

Canada’s mental health and criminal justice systems have long operated in parallel, creating gaps in care for people whose mental health needs intersect with justice involvement. In 2026, the Mental Health Commission of Canada released *Finding New Pathways: An Action Plan for Criminal Justice and Mental Health in Canada*, the country’s first national action plan on this intersection. This policy brief uses the action plan to examine four cross-jurisdictional integration challenges for health system leaders: correctional healthcare governance, mental healthcare continuity inside correctional institutions, information sharing across sectors, and discharge planning at release. Progress requires coordinated action across health, justice, and housing sectors, shaped by people with lived and living experience.

## 1 Introduction

Canada’s mental health and criminal justice systems have developed largely in parallel, leaving people who come into contact with the law during a mental health crisis without adequate clinical and social support, and routing unmet needs into criminal justice channels. The criminal justice system encompasses police, courts, probation and parole, and correctional facilities, all operating under a public safety mandate. The forensic mental health system intersects with criminal justice but is structured differently, assessing and treating people found Unfit to Stand Trial or Not Criminally Responsible on Account of Mental Disorder under separate legislation, review boards, hospitals, and community services (1).

Most justice-involved people with mental illness never receive a forensic designation (2). They move through police contact, courts, custody, and community supervision without the clinical oversight a forensic pathway would provide, and often without adequate mental health support along the way (3). As Szigeti observes, the two systems often serve the same people, and who receives a forensic designation “is so often something that happens by chance and based on factors well outside of … personal control” (4, p. 25).

As of the most recent prevalence data available, 73% of federally incarcerated men and 79% of federally incarcerated women met criteria for one or more mental illnesses, rates that are two to three times higher than in the general population (5–7). A study of admissions to provincial prisons in British Columbia found that the proportion of people with any mental health or substance use need increased from 61% in 2009 to 75% in 2017, with co-occurring disorders doubling over that time (8).

In response to these longstanding challenges, the Mental Health Commission of Canada (MHCC) released *Finding New Pathways: An Action Plan for Criminal Justice and Mental Health in Canada* in 2026. It is Canada’s first national action plan devoted to the intersection of mental illness and the justice and forensic systems. This policy brief uses the action plan as a policy framework and focuses on four integration challenges where health system leaders have direct levers for change: governance of correctional healthcare, continuity of mental healthcare inside correctional institutions, information sharing across sectors, and discharge planning and transitions at release.

## 2 Policy options and implications

*Finding New Pathways* contains 68 evidence-informed recommendations across three strategic pillars: care before, during, and after justice involvement. For policy makers and health system leaders, the central implication is that mental healthcare cannot be improved through isolated correctional reforms alone; progress depends on integrated governance, service delivery, information sharing and systems, and release planning across health, justice, housing, and community sectors. The action plan was informed by a four-phase development process that engaged hundreds of contributors. The process was grounded in two published evidence syntheses, including a rapid scoping review on the mental health needs of justice-involved people (3) and a scoping review of psychological health and safety practises to support criminal justice workers (9). It was also informed by an internal evidence synthesis to guide the policy framework and recommendations, a national survey of nearly 1,200 MHCC network members, and dedicated engagement sessions with First Nations, Inuit, and Métis contributors. People with lived and living experience shaped all stages of the work.

The action plan addresses the social determinants of health, pre-justice crisis response and diversion, continuity of mental healthcare across correctional and community settings, and post-release discharge planning and supports. It also addresses the psychological health and safety of justice system workers, noting that the conditions harming people in the system and those who work within it are connected problems that research and policy have yet to take seriously (9, 10). The action plan attends throughout to the distinct needs of priority populations, including First Nations, Inuit, and Métis peoples, African, Caribbean, and Black people, women, people with intellectual and developmental disabilities, and 2SLGBTQ + people, with anti-racist, trauma-informed, and culturally safe approaches.

Prevention and diversion are a substantial part of *Finding New Pathways*. The plan identifies specialised mental health crisis intervention teams, crisis response centres with no-refusal policies, expanded pre-trial diversion programmes, sustained funding for mental health courts, and community justice centre models that co-locate legal processes with mental health, housing, and social supports. These mechanisms already exist in Canada but are chronically underfunded and unevenly available. For example, the action plan highlights Ottawa’s Alternate Neighbourhood Crisis Response (ANCHOR) programme as a non-police, community-based response for people experiencing mental health or substance use crises (11), and Toronto’s Gerstein Crisis Centre, which provides community-based services through a no-refusal policy to ensure individuals are not turned away during a crisis (12). The plan also points to Ontario Justice Centres, which integrate court processes with treatment, support services, and other wraparound interventions to address underlying causes of justice involvement (13). These examples demonstrate how diversion and community-based responses can improve access to care and reduce reliance on the criminal justice system. The more effectively people are supported before and at first contact, the less pressure falls on the institutional systems examined here.

Nine cross-cutting considerations run through all three pillars, including the social determinants of health, stigma and discrimination, poor information sharing, system-related trauma, and the distinct needs of priority populations and of victims and survivors of crime. Decades of inattention to these issues have constrained system transformation, and addressing them requires dedicated leadership and integration across sectors and ministries at all levels of government.

The action plan highlights four integration challenges that health system leaders can influence. What follows is necessarily selective given the length and scope of this policy brief. The complete set of recommended actions is in the action plan (14). The sections that follow examine each challenge, whilst Table 1 sets out the corresponding recommended actions in detail.

**Table 1:** Actionable recommendations for the four integration challenges.

Integration challenge	Recommended actions	Lead actors and levers
Governance of correctional healthcare	Transfer correctional healthcare delivery, including mental health and psychiatric care, from justice and corrections ministries to health ministries in remaining jurisdictions. Pursue legislative reform and strengthened oversight. Establish agreements between Correctional Service Canada and provincial/territorial (P/T) health systems to deliver fully integrated services.	Federal and P/T ministries of health; Correctional Service Canada; P/T ministries of justice and public safety; P/T health authorities; independent oversight bodies
Continuity of mental healthcare inside institutions	Organise mental healthcare in correctional settings as a continuum of care, with timely screening, individualised treatment planning, culturally safe services, and clear pathways between primary, intermediate, acute, and community care. Expand intermediate care capacity at all security levels and jurisdictions, with clear standards differentiating therapeutic mental health units from isolating conditions of confinement. Use Section 29 of the CCRA as a model for standardised P/T transfer frameworks, supported by improved training and partnership agreements between correctional facilities and community psychiatric hospitals, and by sufficient hospital and community capacity to support timely transfers and continuity of care.	Correctional health services and health leaders; Correctional Service Canada; P/T ministries of health; P/T ministries of justice and public safety; community-based services and mental health hospitals
Information sharing across sectors	Develop a national framework for secure, privacy-compliant information sharing across community and institutional providers. Expand use of electronic health records in correctional settings. Establish data-sharing agreements among corrections, forensic mental health, and community services. Update information-sharing agreements with Indigenous communities to honour data sovereignty principles, including OCAP® (ownership, control, access, and possession) for First Nations people, OCAS (ownership, control, access, and stewardship) for Manitoba Métis people, and Qaujimajatuqangit (“that which Inuit have always known to be true”) for Inuit people. Centre information sharing on equity and individual care planning.	Federal and P/T governments; corrections; forensic and community mental health services; Indigenous governance bodies
Discharge planning and transitions at release	Assign case managers within 6 weeks of incarceration where possible, with clear discharge plans at least 3 weeks before release for sentenced individuals. Provide immediate prescription coverage upon release, with no waiting periods and reliable access to medications over at least the first 30 days. Implement and scale up transitional and supportive housing and Housing First models without sobriety or treatment compliance requirements. Reform legislation to reduce the housing-related consequences of criminal records.	Federal and P/T ministries of health, justice, and public safety; correctional and forensic mental health services; community mental health providers; housing sector; P/T and federal legislators

### 2.1 Governance: transferring correctional healthcare delivery to health ministries

Among the action plan’s most ambitious recommendations is the transfer of correctional healthcare delivery, including mental health and psychiatric care, out of justice and corrections ministries and into health ministries. This governance question is foundational because it shapes clinical independence, accountability, workforce conditions, data systems, and continuity with community care. Six provinces have transferred or are in the process of transferring correctional healthcare to their ministries of health. In the remaining jurisdictions, health services in provincial and territorial (P/T) facilities and in federal institutions under Correctional Service Canada remain under non-health ministries. Federally, the Corrections and Conditional Release Act (CCRA) establishes a statutory duty to provide essential healthcare in conformity with professionally accepted standards (15), whereas P/T facilities are governed by a patchwork of correctional, health, and mental health legislation that differs across jurisdictions and does not provide the same uniform national standard.

Evidence from British Columbia suggests that the governance shift improved provider job satisfaction and increased post-release health service uptake (16) and was associated with reductions in overdose and all-cause mortality (17). People released after the change were more likely to access community healthcare and substance use services (17). The World Health Organisation’s Health in Prisons Programme recommends the health ministry model to reduce inequities and improve outcomes (18).

The gap between the federal legal obligation under the CCRA and what happens in practise has been documented repeatedly by the Office of the Correctional Investigator, the College of Family Physicians of Canada, and other organisations (19–23). Healthcare managed within a corrections mandate runs into fundamental conflicts. When security needs are prioritised over treatment needs and providers work within a structure that prioritises institutional management over therapeutic outcomes, clinical independence is compromised (15, 24). Ontario’s Expert Advisory Committee on Health Care Transformations in Corrections reached similar conclusions, recommending in 2019 that correctional health services be brought under health ministry oversight (25). However, governance reform alone does not guarantee access to evidence-based care. Broader policy decisions also shape service availability, particularly for equity-deserving populations, raising ethical questions and influencing outcomes for people involved in the justice system.

### 2.2 Care continuity: building a mental health continuum inside institutions

Mental healthcare inside correctional settings depends on a functional continuum of care. Governance reform can create the conditions for that continuum, but it does not by itself ensure that people can move between primary services, intermediate care, intensive psychiatric treatment, and diversion to community hospitals when acute needs arise. The action plan addresses each step and identifies where the system falls short. Correctional facilities continue to face difficulties in providing consistent and equitable mental health services at the primary care level (26). Primary care should be the first step in a stepped-care model. The Stepped Care 2.0 model and the international Screening, Triage, Assessment, Intervention, and Reintegration (STAIR) framework provide evidence-informed structures for organising mental healthcare in correctional settings as a pathway with connected, seamless, measurable steps (27, 28). The STAIR framework shows promise as a model for correctional mental healthcare in Canada. The Forensic Early Intervention Service (FEIS), launched at the Toronto South Detention Centre in 2015 and subsequently expanded to the Vanier Centre for Women, operationalizes the framework through a structured process of screening, triage, assessment, intervention, and reintegration planning for remanded individuals with forensic mental health needs (29).

Intermediate care has been developed unevenly across Canada, and in some jurisdictions it has not been developed at all. Correctional Service Canada began introducing Intermediate Mental Health Service Units in 2015, but these units remain unevenly available. They are absent from maximum-security facilities in Quebec, disparities between security levels persist, and the Office of the Correctional Investigator has observed that CSC’s Regional Treatment Centres now function more as intermediate care than as acute psychiatric hospitals (30).

At the intensive care end of the continuum, the underuse of Section 29 of the CCRA emerges as a bottleneck. Section 29 allows federally incarcerated people to be transferred to external psychiatric facilities, but no standardised equivalent exists in P/T custody, where transfer mechanisms, though available in some jurisdictions under P/T mental health legislation, remain inconsistently available and applied. As a result, people in federal and P/T custody face very different prospects for accessing acute mental healthcare governed by different legal frameworks with different degrees of accountability. As the Office of the Correctional Investigator argues, people with acute or complex mental health needs should be transferred under Section 29 to “specialised, external facilities capable of delivering the appropriate level and quality of care” (30, p. 5). Toronto’s FEIS shows how partnerships between correctional facilities and community psychiatric hospitals can be implemented at the P/T level (31).

The action plan also names a tension between the need to divert people with acute mental health and substance use health needs out of prisons and the lack of community capacity to receive them. Community resources are strained, forensic mental health services operate under ongoing capacity and staffing pressures (32), and transfers between facilities can take months or years (4). A critical constraint is supportive housing. Without sufficient supportive housing downstream, forensic beds cannot discharge patients, which blocks transfers from correctional facilities, which in turn prevents intake of new admissions from custody. Bill S-205 proposes to address this through legislation by requiring that anyone in a correctional facility who experiences a disabling mental health issue be transferred to a hospital for care (33). The Office of the Correctional Investigator has made a similar call through policy (30). Progress will likely depend more on hospital capacity, supportive housing, and community-based services than on whether change comes through legislation or policy.

### 2.3 Information sharing: a structural discontinuity

Even where governance and service capacity improve, continuity of care still depends on whether information can be shared safely across the justice-mental health continuum. Poor information sharing disrupts care from intake screening in custody to discharge coordination in the community. Privacy legislation limits access to mental health information and what service providers can act on during crises. Information-sharing protocols and the periodic deletion of mental health records introduce further complications. People often undergo multiple screenings and assessments because systems do not share records, diverting resources from treatment and diversion and eroding continuity of care (14).

Correctional staff and healthcare providers also work under fundamentally different mandates, resulting in mistrust between professionals and ideological friction over whether to prioritise security or care (14). Fragmented and incompatible electronic health records also contribute to the problem. Treatment plans often do not transfer between custodial and community settings, medication continuity is regularly disrupted, and people commonly leave custody without the community care connections reintegration requires (34). For racially marginalised populations, the necessary data to track outcomes and identify structural inequities is often not collected in the first place (14).

Ontario’s use of the VitalHub electronic health record system in provincial correctional facilities shows that health information can be collected and shared across settings to support decision making (35). However, broader implementation has stalled due to limited buy-in and cross-jurisdictional coordination. Expanded information and data sharing also introduces dangerous risks. Health information shared beyond clinical settings can affect employability and other life outcomes for justice-involved people who already face significant structural disadvantages. Information sharing must remain centred on equity and individual care planning to prevent the exacerbation of structural inequities.

### 2.4 Discharge planning and transitions: structural barriers at release

Release from custody is the point at which challenges in governance, care continuity, and information sharing become most visible. Justice-involved people identify release from custody as one of the points where integration breaks down most often (36). Federal and P/T systems differ in discharge planning. People leaving provincial pre-trial custody may be discharged from court without support and may not even return to the same facility to collect belongings, including medications and identification. Release without a formal plan or assured housing is common for P/T sentenced people, and the quality of discharge planning varies by jurisdiction, facility, and available community resources (37, 38). People leaving a federal institution on conditional release, including day parole, are subject to community supervision, and their release plan may include mental health support, though these are not always offered consistently (39). Others are released at sentence expiration with no community support. In a study of people recently released from a provincial gaol in Ontario, Hu et al. (40) asked participants what it was like to leave custody. The title of their paper captures what they heard: “When you first walk out the gates … where do you go?” People often leave correctional and forensic mental health settings without stable housing, medications, appointments with community providers, or continuity of care (40). The Assess, Plan, Identify, and Coordinate (APIC) model, used in Ontario, offers a structured approach to this transition (41). The New Day Programme in Newfoundland, where case managers work both inside prisons and in the community, shows what institution-to-community continuity looks like when built into service design (42).

Forensic systems generally produce more structured pre-discharge planning than correctional releases because review boards must be satisfied that a conditional discharge is necessary and appropriate in light of public safety, a person’s current mental health status, reintegration, and other needs, which in practise often depends on a viable community plan. However, the gap widens at absolute discharge, when formal supervision ends and transition to civil community mental healthcare is not guaranteed. Unable to rely on follow-up in the community, review boards often delay absolute discharge (4), which extends forensic hospitalisation beyond what might be clinically necessary and can even erode positive steps towards recovery for forensic patients, all whilst leaving the underlying continuity problem unresolved.

People leaving custody without follow-up appointments and without access to transitional or supportive housing face a clinical emergency (40). British Columbia’s Community Transition Teams, which provide care coordination and peer support to people released from provincial custody, demonstrate what integrated post-release support looks like when properly resourced (43, 44).

Approximately 30% of people released from federal and P/T correctional institutions in Canada experience homelessness within 2 years of release (45). This reintegration crisis reflects a growing trend across the country, where up to 45% of Canadians report being concerned about their ability to afford stable housing (46). Ontario’s subsidised housing options are limited, with average waitlists of 4 years and longer in some regions (47). For those with ongoing mental health needs, Assertive Community Treatment teams are at capacity and face long waitlists (48), leaving people hospitalised because the housing and mental health supports needed for discharge do not exist (49). Even where housing and mental health services exist, another barrier emerges when community mental health providers in Ontario explicitly refuse patients due to their history of involvement in the forensic mental health system (31). This issue reflects persistent structural stigma for people involved in the criminal justice and forensic mental health systems (50, 51).

Evidence shows that stable housing reduces subsequent justice involvement and supports recovery for people with serious mental illness (52–54). Reintegration therefore depends on coordinated action and integration across health, housing, and criminal justice sectors.

## 3 Actionable recommendations

Table 1 consolidates the recommended actions arising from the four integration challenges, drawing on the action plan’s recommendations and the evidence reviewed above. Together, these actions form a coherent system of reforms and therefore cannot be implemented in isolation. Governance reform can enable continuity, but continuity is only meaningful if it connects people to well-resourced community care. Discharge planning, in turn, depends on the availability of housing, treatment, and social supports available upon release. Implementation will require coordinated action across federal and P/T health, justice, and housing sectors. Throughout, people with lived and living experience must shape the work, as they did during the action plan’s development.

## 4 Conclusion

Keeping mental healthcare governed by corrections ministries limits clinical independence and oversight, makes continuity of care difficult, and leaves people released without clear follow-up or dependable links to health and social services. Poor information sharing compounds these problems at intake, during custody, and at release, whilst barriers to release remain shaped by unavailable community support. These harms disproportionately affect Indigenous Peoples, racialized communities, people with co-occurring disorders, and other priority populations identified in the action plan, who face marginalisation at every stage. Without confronting how that harm is unevenly distributed, integration risks streamlining longstanding failures for the same people.

Critically, implementing the action plan’s recommendations will require major changes in healthcare services alongside reform in criminal justice: expanded primary and secondary care in correctional settings with clear mechanisms for acute care transfers to hospitals, substantially larger investments in community-based mental health and substance use services, and cross-sector integration to connect these elements across federal and P/T systems. This is a complicated undertaking within a federalist system, but the action plan helps to lay out where these systems can start and where they can work together.

Governance failures, gaps in correctional mental healthcare, poor information sharing, and fragmented transitions at points of release have been documented for decades in inquiries and reports, with limited implementation response. For the first time in Canada, *Finding New Pathways* offers a national policy architecture that maps these issues, links them to implementation considerations, clarifies accountability across sectors and actors, and highlights promising practises that serve as templates for other jurisdictions to adapt. Whether the action plan produces these changes will depend on how governments and health system leaders move from mapping these problems to implementing coordinated reforms, and whether implementation is shaped by people with direct experience of these systems.
